# Cell division cycle 42 effector protein 4 inhibits prostate cancer progression by suppressing ERK signaling pathway

**DOI:** 10.17305/bb.2023.9986

**Published:** 2024-08-01

**Authors:** Xiaowen Zhang, Tao Yu, Guojun Gao, Junbao Xu, Ruihui Lin, Zhifang Pan, Jianying Liu, Weiguo Feng

**Affiliations:** 1 School of Life Science and Technology, Weifang Medical University, Weifang, China; 2 Department of Urology Surgery, Affiliated Hospital of Weifang Medical University, Weifang, China; 3 Cancer Center, Shandong Public Health Clinical Center, Shandong, China; 4 Department of Nuclear Medicine, The Third Affiliated Hospital of Shandong First Medical University, Jinan, China

**Keywords:** Cell division cycle 42 effector protein 4 (CDC42EP4), prostate cancer (PCa), extracellular signal-regulated kinase (ERK), proliferation, invasion

## Abstract

Prostate cancer (PCa) is the most common malignancy among men worldwide. The cell division cycle 42 effector protein 4 (CDC42EP4) functions downstream of CDC42, yet its role and molecular mechanisms in PCa remain unexplored. This study aimed to elucidate the role of CDC42EP4 in the progression of PCa and its underlying mechanisms. Bioinformatical analysis indicated that CDC42EP4 expression was significantly lower in PCa tissue compared to normal prostate tissue. Cellular phenotyping analysis suggested that CDC42EP4 markedly inhibited the proliferation, migration, and invasion of PCa cells. Xenograft tumor assays further demonstrated that CDC42EP4 suppressed the growth of PCa cells in vivo. Mechanistically, the study established that CDC42EP4 inhibited the extracellular signal-regulated kinase (ERK) pathway in PCa cells. Additionally, the ERK pathway inhibitor PD0325901 was employed, revealing that PD0325901 significantly nullified the effects of CDC42EP4 on PCa cell proliferation, migration, and invasion. Collectively, our findings demonstrate that CDC42EP4 acts as a critical tumor suppressor gene, inhibiting PCa cell proliferation, migration, and invasion through the ERK pathway, thereby presenting potential targets for PCa therapy.

## Introduction

Prostate cancer (PCa) is recognized as the most common malignant tumor and a leading cause of oncological deaths in men worldwide [1–5]. In the United States alone, an estimated 288,300 men are projected to be diagnosed with PCa, and 34,700 men are expected to die from it in 2023 [6]. The primary treatments for early-stage PCa currently include surgery and androgen deprivation therapy (ADT) [7]. However, a significant number of patients with PCa inevitably progress to castration-resistant PCa (CRPC), which is associated with poor prognosis [8, 9]. Therefore, understanding the pathogenesis of PCa and identifying new therapeutic targets are critically important.

Cell division cycle 42 (CDC42), a key member of the Rho GTPase protein family, plays a role in various cellular functions, including cell proliferation, migration, invasion, and pseudopod formation [10, 11]. CDC42 effector proteins (CEPs) are a group of effector proteins downstream of CDC42, comprising five members: CDC42EP1/2/3/4/5 [12]. CDC42EP4, also known as Binder of Rho GTPases-4, is widely expressed in all human tissues [13, 14]. To date, only a few reports have indicated a strong association between CDC42EP3 and tumorigenesis and progression [15, 16], with no studies exploring the role and mechanisms of CDC42EP4 in tumor progression.

Extensive research has linked epithelial–mesenchymal transition (EMT) with PCa migration and invasion, characterized by changes in gene expression, including E-Cadherin and N-Cadherin. These alterations lead to epithelial cells losing their junctions and apical–basal polarity and undergoing cytoskeletal reorganization [17, 18]. CyclinD1, a key cell cycle regulator, is also involved in cell proliferation [19, 20]. Furthermore, the extracellular signal-regulated kinase (ERK) pathway, a critical component of the mitogen-activated protein kinase (MAPK) pathway [21], has been associated with PCa occurrence, progression, and poor prognosis due to elevated ERK phosphorylation levels [22, 23]. Consequently, exploring the ERK signaling mechanism in PCa tumorigenesis could bring clinical benefits to patients.

The present study aims to elucidate the role of CDC42EP4 in PCa progression and its intrinsic mechanisms, potentially providing new targets for PCa treatment.

## Materials and methods

### Reagents and antibodies

Fetal bovine serum (FBS) and Dulbecco’s modified eagle’s medium (DMEM) were purchased from Thermo Fisher Inc. (Shanghai, China). Anti-CDC42EP4 was purchased from Sigma-Aldrich (MO, USA). Cell counting kit 8 (CCK-8), PD0325901, enhanced chemiluminescence reaction kit (ECL), the secondary antibodies, anti-ERK1/2, anti-p-ERK1/2 (Thr202/Tyr204), anti-E-Cadherin, anti-N-Cadherin, anti-CyclinD1, and anti-β-actin were purchased from Beyotime Biotechnology (Shanghai, China). CDC42EP4 shRNA lentiviral particles and CDC42EP4 lentiviral activation particles were purchased from Santa Cruz (CA, USA).

### Bioinformatical analysis

The expression of CDC42EP4 was analyzed using the UALCAN database (http://ualcan.path.uab.edu/). The effect of CDC42EP4 expression on disease-free survival was examined using the GEPIA database (http://gepia.cancer-pku.cn/). Additionally, the protein expression of CDC42EP4 was verified by the Human Protein Atlas database (http://www.proteinatlas.org/).

### Lentiviral transfection and cell culture

PCa cell lines, including PC3 and 22RV1, were obtained from the Shanghai Cell Bank. Stable sh-CDC42EP4-22RV1 and overexpressing CDC42EP4-PC3 cell lines were constructed by transfecting CDC42EP4 shRNA lentiviral particles and CDC42EP4 lentiviral activation particles, respectively. All cells were cultured in DMEM containing 10% FBS.

### CCK-8 and colony formation assay

In a CCK-8 assay, PC3 and 22RV1 cells, including parental cells and transfected cells (3000 per well), were inoculated separately into 96-well plates and then the relative viability of the cells was assessed at different time points according to the methodology of our previous study [24]. In a cell colony formation assay, as in our previous study, PC3 and 22RV1 cells, including parental cells and transfected cells (200 per well), were seeded separately in 6-well plates and examined for colony formation after 14 days [25].

### Wound healing and invasion assay

In the wound healing experiment, PC3 and 22RV1 cells, including parental cells and transfected cells, respectively, were seeded in 6-well plates, cultured in serum-free medium, and then cell-free gaps were created using a micropipette tip and photographed under the microscope 24 or 48 h later, as described in our previous study [24]. In the invasion experiment, PC3 and 22RV1 cells, including parental cells and transfected cells, were seeded in transwell chambers, respectively. Cells that had invaded the membrane were stained and analyzed using 0.5% crystal violet solution after 24 h of incubation as described previously [26].

### Western blot analysis

In short, in the western blot experiment, proteins were processed by SDS-PAGE and then transferred to a PVDF membrane. Subsequently, the membrane was incubated with primary antibodies, including anti-CDC42EP4 (1:1000), anti-ERK (1:1000), anti-p-ERK (1:1000), anti-E-Cadherin (1:1000), anti-N-Cadherin (1:1000), anti-CyclinD1 (1:1000), anti-β-actin (1:1000), and secondary antibodies (1:1000), and then the protein bands were measured using the ECL kit as described previously [27].

### In vivo assay for tumor growth

Four-week-old male nude mice (BALB/c) were reared for one week and then injected subcutaneously with control and CDC42EP4 low-expression group 22RV1 cells (5×10^6^ cells/mouse), respectively. Tumor growth was monitored every seven days and tumor volume was determined by the formula: 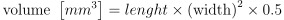. Finally, tumor weight was assessed after the mice were euthanized by carbon dioxide.

### Ethical statement

The animal study was approved by the Animal Ethics Committee of Weifang Medical University (2021SDL507, Weifang, China).

### Statistical analysis

Data were analyzed by independent samples *t*-test or ANOVA using SPSS 19.0 software. The experiment was repeated at least three times. Data were displayed as mean ± SD, with *P* < 0.05 representing statistically significant differences.

## Results

### Bioinformatical analysis of CDC42EP4

**Figure 1.**
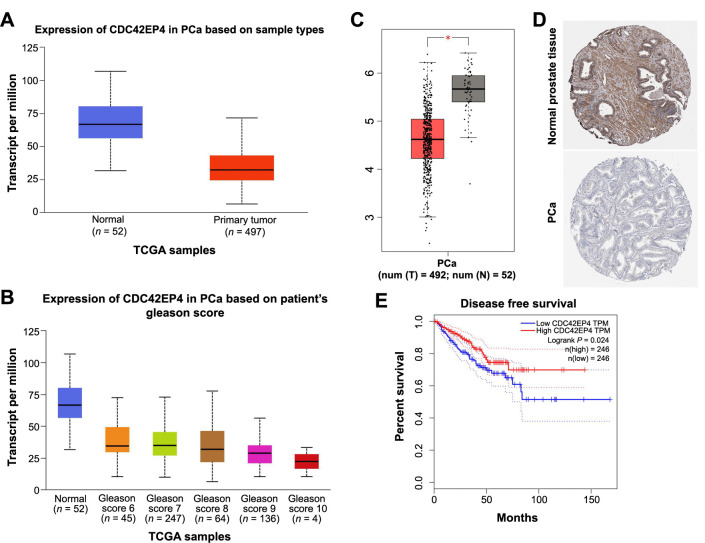
**Bioinformatical analysis of CDC42EP4.** (A and B) The expression of CDC42EP4 from the UALCAN database; (C) The expression of CDC42EP4 from the GEPIA database; (D) The expression of CDC42EP4 from the Human Protein Atlas database; (E) The effect of CDC42EP4 expression on disease-free survival from the GEPIA database. CDC42EP4: Cell division cycle 42 effector protein 4; PCa: Prostate cancer.

The expression and prognosis of CDC42EP4 were analyzed using various bioinformatics tools, including UALCAN, GEPIA, and the Human Protein Atlas database. The analysis revealed that CDC42EP4 expression in PCa tissues was significantly lower compared to normal prostate tissues (Figure 1A, 1C, and 1D). Notably, a strong correlation was observed between the expression of CDC42EP4 and the Gleason grade of PCa, suggesting that CDC42EP4 could serve as a diagnostic marker for PCa (Figure 1B). Moreover, the results indicated that the expression of CDC42EP4 was closely related to the prognosis of PCa (Figure 1E). These findings suggest that CDC42EP4 may play an important role in PCa progression and warrants further investigation.

### CDC42EP4 inhibits the proliferation, migration, and invasion of PCa cells

**Figure 2.**
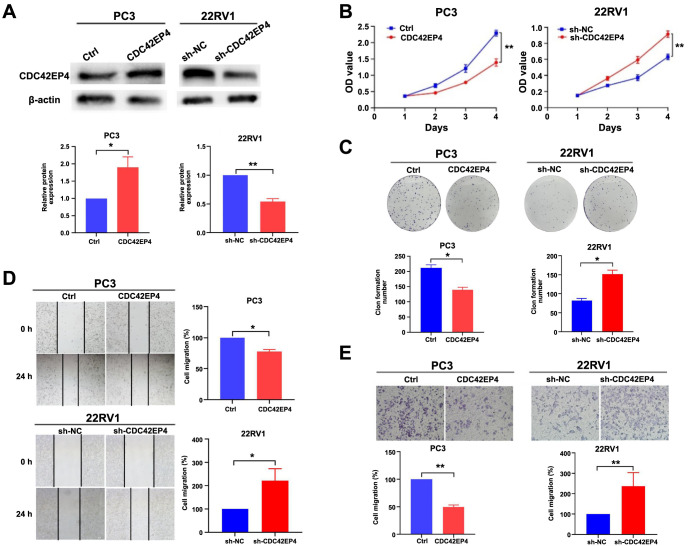
**CDC42EP4 inhibits the proliferation, migration, and invasion of PCa cells.** (A) A construction of CDC42EP4 overexpression and low-expression cell lines; (B) The effect of CDC42EP4 on cell proliferation determined by CCK-8 test; (C) The effect of CDC42EP4 on cell proliferation assessed by colony formation test; (D) The effect of CDC42EP4 on cell migration measured by wound-healing test; (E) The effect of CDC42EP4 on cell invasion tested by transwell assay. *P < 0.05, **P < 0.01. Error bars indicate SE. CDC42EP4: Cell division cycle 42 effector protein 4; PCa: Prostate cancer; CCK-8: Cell counting kit 8; Ctrl: Control.

To investigate the role of CDC42EP4 in PCa, PC3, and 22RV1 cells were utilized to construct CDC42EP4 overexpressing and low-expressing cell lines, respectively. The results demonstrated that CDC42EP4 expression was markedly higher in the CDC42EP4 overexpressing group than in the control group, and CDC42EP4 expression was significantly lower in the sh-CDC42EP4 group than in the control group (*P* < 0.05, Figure 2A). This confirmed the successful construction of CDC42EP4 overexpression and low-expression cell lines.

Subsequently, assays, including CCK-8, colony formation, wound healing, and transwell tests, were performed to evaluate the effects of CDC42EP4 on PCa cell proliferation, migration, and invasion. The CCK-8 and colony formation assays indicated that CDC42EP4 overexpression significantly suppressed the PC3 cell proliferation, whereas CDC42EP4 low expression markedly enhanced the proliferation of 22RV1 cells (*P* < 0.05, Figure 2B and 2C). Likewise, wound-healing and transwell assays revealed that cell migration and invasion capacity were inhibited by CDC42EP4 overexpression, while its low expression promoted the migration and invasion capacities of the cells (*P* < 0.05, Figure 2D and 2E). Collectively, these results demonstrate that CDC42EP4 inhibits the proliferation, migration, and invasion of PCa cells.

### CDC42EP4 inhibits PCa cell growth in vivo

**Figure 3.**
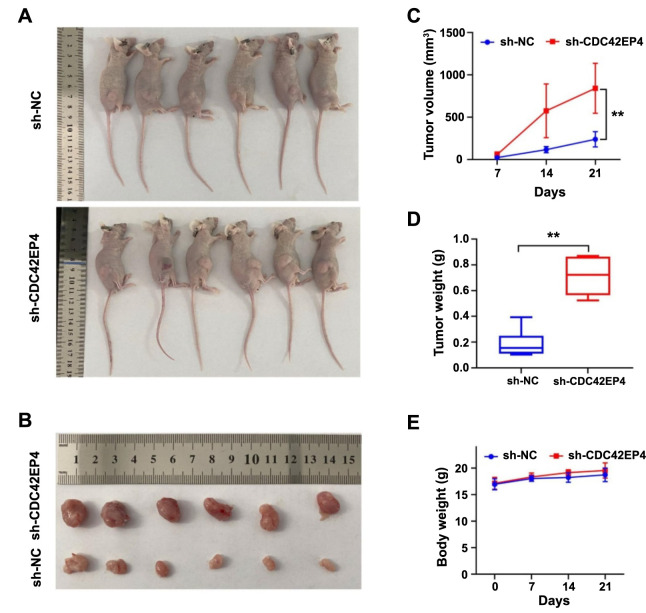
**CDC42EP4 inhibits PCa cell growth in vivo.** The sh-NC and sh-CDC42EP4 22RV1 cells were injected subcutaneously into nude mice, and the tumor volume was measured at various time points. The nude mice were euthanized by carbon dioxide and the tumor weight was analyzed. (A) The nude mice; (B) The tumor of nude mice; (C) The tumor volume of nude mice; (D) The tumor weight of nude mice; (E) The body weight of nude mice. **P < 0.01. Error bars indicate SE. CDC42EP4: Cell division cycle 42 effector protein 4; PCa: Prostate cancer.

To validate the inhibitory effect of CDC42EP4 on PCa cell growth, xenograft tumor assays were conducted (Figure 3A and 3B). The results showed that 22RV1 cells with low CDC42EP4 expression were more proliferative. The tumor volume was remarkably higher in the sh-CDC42EP4 group than in the sh-NC group (841.7 vs 239.3 mm^3^, *P* < 0.01, Figure 3C). Similarly, the tumor weight was significantly increased in the sh-CDC42EP4 group relative to the sh-NC group (0.71 vs 0.19 g, *P* < 0.01, Figure 3D). However, there was no significant change in the body weight of the mice (Figure 3E). These findings confirm that CDC42EP4 inhibits PCa cell growth in vivo.

**Figure 4.**
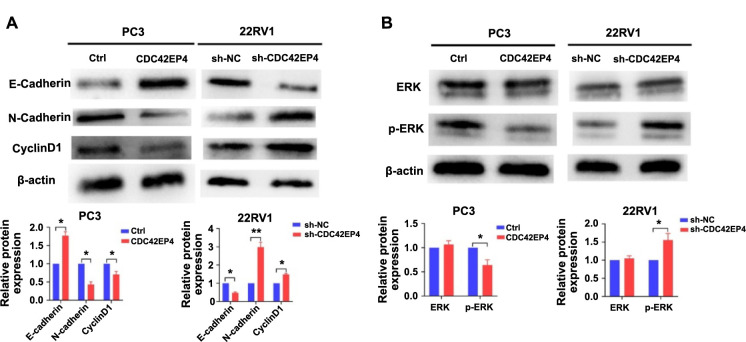
**CDC42EP4 regulates markers of malignant phenotype and ERK pathway.** (A) The expression of E-Cadherin, N-Cadherin, and CyclinD1 measured by western blot; (B) The expression and phosphorylation of ERK determined by western blot. *P < 0.05. Error bars indicate SE. CDC42EP4: Cell division cycle 42 effector protein 4; ERK: Extracellular signal-regulated kinase; Ctrl: Control.

### CDC42EP4 regulates markers of malignant phenotype and ERK pathway

Considering that the markers of malignant phenotypes, such as E-Cadherin, N-Cadherin, and CyclinD1 are strongly associated with PCa progression, an investigation was conducted to determine whether CDC42EP4 regulates the expression of these molecules. The western blot result showed that CDC42EP4 overexpression significantly upregulated the expression of E-cadherin and downregulated the expression of N-Cadherin and CyclinD1 in PC3 cells. In contrast, low CDC42EP4 expression significantly attenuated the expression of E-cadherin, while elevating the expression of N-Cadherin and CyclinD1 in 22RV1 cells (*P* < 0.05, Figure 4A). These data demonstrate that CDC42EP4 regulates markers of malignant phenotype in PCa cells.

Previous studies established that the ERK pathway is strongly correlated with PCa progression [21, 23]. Therefore, the potential regulation of the ERK pathway by CDC42EP4 was also assessed. As shown in Figure 4B, the ERK expression remained unchanged, but its phosphorylation level was significantly lower in the CDC42EP4 overexpression group and higher in the sh-CDC42EP4 group relative to the control group (*P* < 0.05). These results proved that CDC42EP4 inhibits the ERK pathway in PCa cells.

### CDC42EP4 inhibits PCa progression through ERK pathway

**Figure 5.**
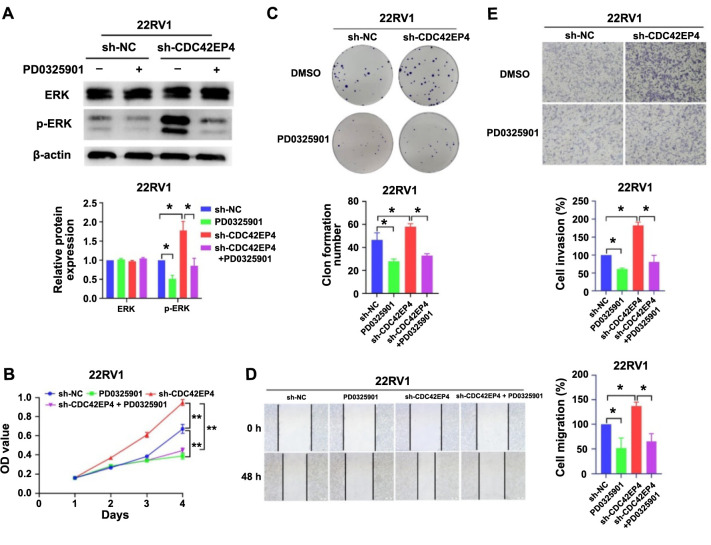
**CDC42EP4 inhibits PCa progression through theERK pathway.** The 22RV1 cells in sh-NC and sh-CDC42EP4 groups were treated with or without PD0325901 (10 nM) for 24 h. (A) The expression and phosphorylation of ERK tested by western blot; (B and C) The cell proliferation determined by CCK-8 and colony formation assays; (D) The cell migration assessed by wound-healing test; (E) The cell invasion measured by transwell test. *P < 0.05, **P < 0.01. Error bars indicate SE. CDC42EP4: Cell division cycle 42 effector protein 4; PCa: Prostate cancer; ERK: Extracellular signal-regulated kinase; CCK-8: Cell counting kit 8.

To determine whether CDC42EP4 inhibits PCa progression via the ERK pathway, the ERK pathway inhibitor PD0325901 was utilized. Western blot analysis revealed that PD0325901 significantly inhibited the ERK phosphorylation promoted by low CDC42EP4 expression in 22RV1 cells (*P* < 0.05, Figure 5A). The data from CCK-8, colony formation, wound healing, and transwell assay tests suggested that PD0325901 significantly counteracted the effect of CDC42EP4 on the proliferation, migration, and invasion of 22RV1 cells (Figure 5B–5E, *P* < 0.05). Together, the results showed that CDC42EP4 inhibits the progression of PCa through the ERK pathway.

## Discussion

In this study, we discovered that CDC42EP4 expression was significantly lower in PCa tissues compared with normal prostate tissues. Phenotypic experiments on cells demonstrated that CDC42EP4 remarkably inhibited the proliferation, migration, and invasion of PCa cells. Additionally, xenograft tumor assays revealed that CDC42EP4 suppressed the growth of PCa cells in vivo. Our mechanistic studies demonstrated that CDC42EP4 impedes PCa progression through the ERK pathway.

CDC42EP4, also known as the Rho GTPases-4 binder, belongs to CEPs and is widely expressed in all human tissues [13, 14]. In recent years, limited reports have shown that CDC42EP3, a member of the same family as CDC42EP4, is highly expressed in gastric cancer [16], gliomas [15], and colorectal cancer [28]. However, the link between CDC42EP4 expression and cancer remains unreported. Our findings suggest that the downregulation of CDC42EP4 in PCa could serve as a diagnostic and prognostic marker, pointing to its potential role as a key gene in PCa progression.

To date, the role of CDC42EP4 in tumor progression remains unexplored. Limited studies showed that CDC42EP4 phosphorylation promoted breast cell motility [12]. Others have reported that CDC42EP3 promoted cell proliferation, migration, and inhibited apoptosis in gastric cancer, gliomas, and colorectal cancer [15, 16, 28]. In this study, cell function analysis revealed that CDC42EP4 significantly suppressed PCa cell proliferation, migration, and invasion. Meanwhile, xenograft tumor assay also confirmed that CDC42EP4 inhibited PCa cell growth in vivo. In addition, it has been reported that E-Cadherin and N-Cadherin, the marker of EMT, are closely associated with tumor invasion [29, 30], and CyclinD1 with tumor proliferation [31]. Our data showed that PCa cells overexpressing CDC42EP4 exhibited high levels of E-cadherin and low levels of N-Cadherin and CyclinD1, suggesting that CDC42EP4 may impair the proliferative and invasive properties of PCa cells. These in vitro and in vivo findings propose that CDC42EP4 may act as a tumor suppressor gene, offering a potential therapeutic target for PCa.

The ERK pathway is a well-known oncogenic signaling pathway involved in the development and progression of various tumors, including lung cancer [32], gastric cancer [33], PCa [22], breast cancer [34], ovarian cancer [35], and colorectal cancer [36]. Multiple studies have also shown that the ERK pathway is involved in a variety of cellular phenotypes of PCa, including cell proliferation, migration, and invasion [21, 23, 37]. Therefore, we hypothesized that CDC42EP4 might inhibit PCa tumorigenesis via the ERK pathway. Our data showed that CDC42EP4 suppresses ERK phosphorylation, suggesting its inhibitory effects on the ERK pathway in PCa cells. In addition, our results further proved that PD0325901, an ERK pathway inhibitor, was capable of abrogating the regulatory effects of CDC42EP4 on cell proliferation, migration, and invasion of PCa. Collectively, our findings suggest that CDC42EP4 inhibits PCa cell proliferation, migration, and invasion largely via the ERK pathway. These results enhance our understanding of PCa development and offer a promising strategy for intervening in PCa progression. However, the mechanisms by which CDC42EP4 regulates the ERK pathway remain to be elucidated in further research.

## Conclusion

Our results collectively indicate that CDC42EP4 plays a crucial role as a tumor suppressor gene, significantly inhibiting PCa cell proliferation, migration, and invasion through the ERK pathway. This discovery positions CDC42EP4 as a potential target for PCa treatment strategies.

## Data Availability

The datasets used and/or analyzed during the current study are available from the corresponding author on reasonable request.
